# The Atlanta Meeting of the Medical Association of Georgia

**Published:** 1905-05

**Authors:** 


### *EDITORIAL NOTES AND COMMENTS.*

The Business office of THE JOURNAL-RECORD is No. 1007-1008 Century Building.

The Editorial office is 318-319-320 The Prudential.

Address all Business communications to Dr. M. B. Hutchins, Mgr.

Make remittances payable to THE ATLANTA JOURNAL-RECORD OF MEDICINE.

On matters pertaining to the Editorial and Original Communications address Dr Bernard Wolff, Atlanta.

Reprints of original articles will be furnished at cost price. Requests for the same should always be made *on the manuscript.*

We will present, post-paid, on request, to each contributor of an original article, twenty (20) marked copies of THE JOURNAL-RECORD containing such article.

### THE ATLANTA MEETING OF THE MEDICAL ASSOCIATION OF GEORGIA.

THE fifty-sixth annual meeting of the Medical Association of Georgia was held in the ballroom of the Kimball House, Atlanta, April 19, 20 and 21. The meeting was presided over by the President, Dr. William Perrin Nicolson, and was opened with prayer by the Rev. Millard.

The enrollment was unusually large, not only on account of the important matters to be considered, but also the additional attractions offered the medical man by the meeting of the American Anti-Tuberculosis League, which immediately preceded that of the State Medical Association.

The meeting was, perhaps, the most notable and important in the history of the Association, as the plan for the adoption of the new constitution was to be passed upon. This measure contemplated so radical a change in the existing condition of the Association as practically to recreate it and to establish a regime so different, so novel, and so obliterative of the old, that an interest was aroused which was almost unprecedented. Each member had a strong sense of personal implication and an equally strong desire to be present and throw his weight in one direction or the other.

There seemed to be considerable equanimity of opinion as to the desirability, even the urgent necessity, of effecting a unification of the medical profession in the State of Georgia. Irritated almost beyond endurance by the terrible infliction upon the State of quackery, by the supineness and lethargy of the legislative bodies in the enactment of sanitary and medical laws, by the isolation and loneliness of the little groups of workers for the advancement of the people's good through the medical profession, and in general by its utter lack of power and influence through the looseness of its alignment, the members of the Association were eager to effect a compact organization which promised relief from this galling situation and a means of elevating the profession to that position which the character of its members so justly entitled it. But in the minds of some the buoyancy of hope was darkened by the suspicion that sinister and ulterior motives lurked back of this promise, that is was merely a scheme to further the ambitions of a few; that the reorganization plan was at the bottom designed as a merger in the interest of political graft; that local interests were to be tampered with and controlled by an alien and unfriendly body. These objections were met by the advocates of the plan with the assertion that they were untenable and ridiculous, dictated by jingoism and sectional prejudice; that it was intended solely for the best interest of the Association, and that adopting it the State lost not one whit of its independence, but on the contrary, vastly increased its power, influence and efficiency; that once adopted it was not binding in itself, nor was it a compact with any other organization or group of organizations, and that it could be altered, amended, or abrogated at the will of the Association. It was for an adjustment of these conflicting views that a special session was held on the night of April 20.

The auditorium of the Marist College was crowded with interested and expectant listeners.

The report of the Committee on Reorganization was read by its Chairman, Dr. F. W. McRae. It included a reading of the proposed new constitution. The report was then declared open for discussion. Dr. J. C. Olmstead and Dr. W. F. Westmoreland spoke at length in favor of adopting the report. They were followed by Dr. Chas. Hicks, whose opposition to the plan of reorganization was well known, and whose zeal, sincerity and rugged strength gained for him so large a personal following as to favor a forecast that his faction would prevail; and Dr. T. M. McIntosh, of Thomasville. Both of these gentlemen presented elaborate arguments against the adoption of the new constitution.

The discussion then became general, and finally a vote on the motion to adopt the report of the Reorganization Committee was taken. The vote resulted as follows: Affirmative, 134; negative, 111. The Chairman declared the motion carried. Much confusion then ensued. Dr. Hicks appealed from the decision of the Chair, maintaining that a two-thirds vote was necessary for the motion to carry. The Chairman ruled that a majority only was necessary.

Dr. Westmoreland, having with much difficulty secured a hearing, moved that a committee be appointed consisting of two from each side and that the question of the correctness of the chair's position be submitted to them for adjudication, the opinion of a skilled parliamentarian to be secured should the committee fail to arrive at a conclusion. This motion was carried and the meeting adjourned. The committee appointed consisted of F. W. McRae, W. F. Westmoreland, J. B. Morgan, Chas. Hicks.

The committee shortly before one o'clock on the following day reported that without any outside assistance they found that the ruling of the Chair was correct. The report was signed by Drs. Morgan, Westmoreland and McRae, Chas. Hicks dissenting. The new constitution was therefore adopted and declared immediately operative. When this action had been taken, Dr. Hicks rose and tendered his resignation as a member of the Association, stating that he could not agree with the ruling of the Chair, and that he did not desire to be a source of discord in the Association. There was a strong sentiment against accepting Dr. Hick's resignation. and the suggestion to him that he reconsider his action met with hearty applause. This was further attested by the presentation to Dr. Hicks by some of his admirers during the afternoon session of a handsome gold watch suitably engraved. After the adoption of the new constitution the Association proceeded to the election of officers, which resulted as follows: President, W. Z. Holladay, Augusta; First Vice-President, R. P. Izlar, Waycross; Second Vice-President, C. F. Nolan, Marietta; H. F. Harris, delegate to American Medical Association. The councilors were elected by the members of the Association from the eleven congressional districts, each district electing its own representative: First district, J. S. Howkins, Savannah; Second district, W. L. Davis, Albany; Third district, R. E. L. Barnum, Richland; Fourth district, W. L. Fitz, Carrollton; Fifth district, E. C. Davis Atlanta; Sixth district, M. A. Clark, Macon; Seventh district, A. T. Calhoun, Cartersville; Eighth district, S. C. Benedict, Athens; Ninth district, W. V. Hardman, Commerce; Tenth district, W. W. Pilcher, Warrenton; Eleventh district, J D. Herman, Eastman.

Into the hands of the aforementioned the task of organizing the county medical societies, the prime object of the new system, will be entrusted. The task is a simple one in some districts where local medical bodies already exist. It will doubtless prove a very difficult one in some portions of the States where there are small or no nuclei upon which to establish a unit. It is, however, in just such localities that the greatest results are looked for and the test will be heaviest upon the weakest side. The councilors doubtless realize this obvious circumstance and will rise valorously to the occasion.

It is evident that the Association for the success of the newly adopted plan must exemplify one of the trite maxims of our government, "united we stand, divided we fall." Factional spirit will beget discord and tend to enfeeblement and ultimate disruption. It is the accepted time to subordinate individual differences in the demand for the general good. The smarting of defeat often leads to ill-advised and impetuous action. In this instance reflection will show plainly that intestine strife but serves to weaken the barrier that is erected for the protection of the profession and that if the salutary effects of a united profession are to be enjoyed they must be obtained by an offensive and defensive alliance that is not only united in name but in fact.

